# How Did You Sleep Tonight? The Relevance of Sleep Quality and Sleep–Wake Rhythm for Procrastination at Work

**DOI:** 10.3389/fpsyg.2021.785154

**Published:** 2022-02-28

**Authors:** Tabea Maier, Jana Kühnel, Beatrice Zimmermann

**Affiliations:** ^1^Department of Occupational, Economic, and Social Psychology, Faculty of Psychology, University of Vienna, Vienna, Austria; ^2^Department of Work and Organizational Psychology, Faculty of Engineering, Computer Science and Psychology, Institute of Psychology and Education, University of Ulm, Ulm, Germany

**Keywords:** procrastination, sleep quality, chronotype, summer time, shift to daylight saving time, self-regulation

## Abstract

Recent studies have highlighted the relevance of sleep for procrastination at work. Procrastination at work is defined as the irrational delay of the initiation or completion of work-related activities. In line with recent studies, we offer a self-regulation perspective on procrastination. We argue that procrastination is an outcome of depleted self-regulatory resources and that the restoration of self-regulatory resources during high-quality sleep at night would prevent procrastination.

**Aims:**

In an attempt to further develop this line of research, the current study aimed to achieve a broader understanding of the relevance of sleep and circadian rhythm for procrastination. Therefore, we explored the effect of sleep quality on procrastination for different chronotypes. We also considered the shift to daylight saving time as a phenomenon that aggravates circadian misalignment and thereby later chronotypes' dependence on high-quality sleep. Specifically, we hypothesized that compared to employees with an earlier chronotype (morning types), employees with a later chronotype (evening types) are more dependent on good sleep at night to prevent procrastination the next day. This effect would be especially pronounced after the shift to daylight saving time.

**Methods:**

For this repeated-measures study, participants were 101 full-time employees. They completed a general questionnaire and day-specific questionnaires on the Monday before and the Monday following the shift to daylight saving time.

**Results:**

The multilevel analyses showed that employees procrastinated less on days following nights during which they slept better and that later chronotypes experienced more procrastination than earlier chronotypes. Our findings also supported the hypothesis that the relationship between sleep quality and procrastination is stronger for later chronotypes compared to earlier chronotypes on the Monday following the shift to daylight saving time. In other words, the lower the sleep quality of later chronotypes during the previous night, the more they procrastinated on the Monday following the shift to daylight saving time.

**Discussion:**

Our findings further corroborate the existing findings on the relevance of sleep and chronotype for well-being and performance at work.

## Introduction

“Never put off till tomorrow what you can do the day after tomorrow.” This quote by Mark Twain addresses the phenomenon of procrastination. Procrastination is defined as the tendency to delay the initiation or the completion of activities (Lay, 1986; Howell et al., 2006; Steel, 2007). According to Steel (2007), procrastination reflects an intention–behavior gap. Specifically, people “intend… to do something, but put… it off despite expecting that it will yield negative consequences” (Kühnel et al., 2018b). Procrastination carries the risk of detrimental consequences at work, including strain and poor performance (Steel, 2007; Kim and Seo, 2015), failure to meet deadlines (van Eerde, 2003), risking the success of projects (Gersick, 1989), problems in social relationships (Küchler et al., 2019), and lost productivity (Stead et al., 2010; Beutel et al., 2016).

From the theoretical perspective of self-regulation, procrastination is considered an indicator of failed self-regulation (Ferrari, 2001; Kühnel et al., 2016; van Eerde, 2016). Self-regulation covers the autonomous regulation of goal-directed behavior, which includes adapting thoughts, feelings, desires and actions to personally relevant goals (Ridder and Wit, 2006; Vohs and Baumeister, 2011; Kühnel et al., 2016). Self-regulatory resources are necessary for individuals to regulate and initiate action at work. When an individual has low self-regulatory resources, focus on work tasks is difficult to maintain, and the individual will be more likely to choose pleasurable alternatives. In other words, when self-regulatory resources are low, the individual's action initiation is impeded and procrastination occurs (Tice and Baumeister, 1997). Therefore, restoring self-regulatory resources during non-work periods is a prerequisite for an individual's ability to initiate action at work (Kazén et al., 2008; Kühnel and Sonnentag, 2011).

**Figure 1:**
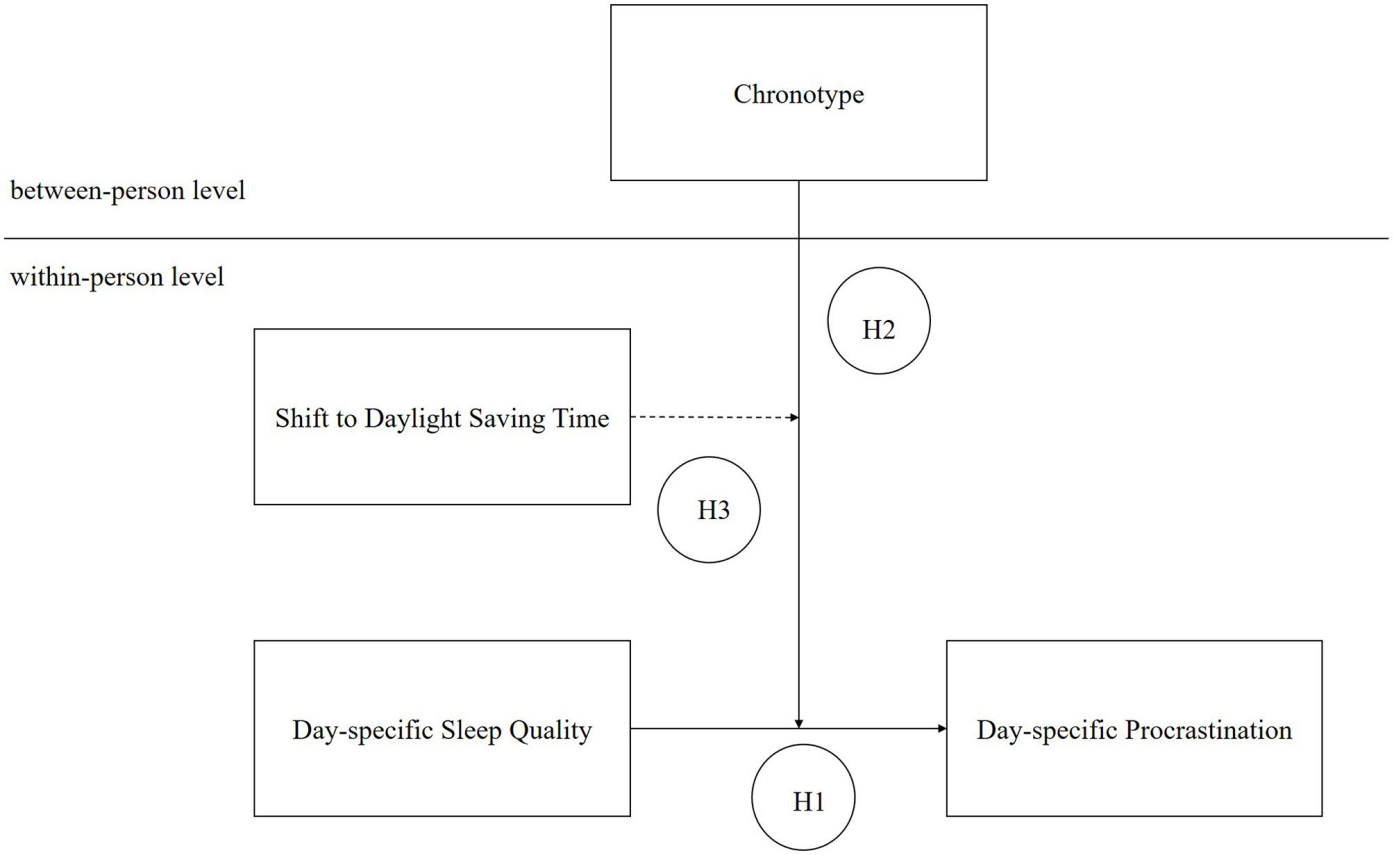
Conceptual model of the current study. Within-person variance (Level-1 variance) captures the variation in variables from day to day within persons and between-person variance (Level-2 variance) captures the variation in variables between persons.

An important non-work period during which self-regulatory resources can be restored is sleep. Recent studies have shed light on the relevance of day-specific sleep quality and people's preferred sleep–wake rhythm (chronotype) for procrastination at work. In line with Baumeister et al.'s (2000) work, Kühnel et al. (2016) showed that high-quality sleep at night restores self-regulatory resources, thereby preventing procrastination the next day. The research into procrastination at work, as well as its relationship with sleep at night, has recently begun to gain attention. To gain further insights into this relationship and contribute to this stream of research, this study aimed to achieve a broad understanding of the relevance of sleep and circadian rhythm for procrastination. This led to three specific aims. First, we sought to replicate the negative relationship between sleep quality and procrastination. Second, we aimed to clarify the role of individually-preferred sleep–wake rhythms in the relationship between sleep and procrastination, and thereby answer the question of whether some people are more dependent on good-quality sleep than others. Third, we focused on the shift to daylight saving time (DST) as a potential promoter of procrastination. The shift to DST is a phenomenon that should aggravate circadian misalignment, and thereby, later chronotypes' dependence on high–quality sleep (Marcus and Schuler, 2004). Figure 1 demonstrates the conceptual model for this study and summarizes the hypotheses we derived from the theoretical perspective of self-regulation.

### Sleep Quality and Procrastination

All activities that require self-regulation draw on a resource that is not infinite (Baumeister et al., 1998). When one's self-regulatory resources are depleted (Marcus and Schuler, 2004), it becomes more difficult to resist dysfunctional or impulsive actions, and procrastination may occur. Recovery, meanwhile, which is characterized by replenishment of the resources necessary for self-regulation (Binnewies et al., 2009), should counteract the occurrence of procrastination. Sleep is a recovery process that ensures restoration of resources that are necessary for self-regulatory functioning the next day (e.g., Baumeister et al., 2000; Barber et al., 2010). Barnes et al.'s (2011) research emphasized the role of sleep *quality*, stating that it provides an important foundation for an individual's self-regulation ability. According to Barnes et al. (2011) poor sleep minimizes and high-quality sleep maximizes self-regulatory resources. This is in line with previous studies showing that high-quality sleep at night restores self-regulatory resources and thus prevents procrastination the next day (Kühnel et al., 2016). Therefore, we assume that people will procrastinate less after nights characterized by better sleep quality compared to nights characterized by lower sleep quality. Consequently, the first hypothesis of this study is that day-specific sleep quality negatively predicts procrastination the next day.

### Chronotype and Procrastination

Our study aimed to address the question of whether some people are more dependent on good-quality sleep to prevent procrastination than others. Studies have highlighted that interindividual differences in biologically preferred sleep–wake rhythm (chronotype) are associated with self-regulation and therefore play a role in procrastination (Díaz-Morales et al., 2008; Digdon and Howell, 2008; Hagger, 2010). The individual characteristic chronotype is normally distributed in the population (Roenneberg et al., 2003). Earlier chronotypes (morning types, or “larks”)—who prefer to go to sleep earlier in the evening and wake up earlier in the morning—sit at one end of the continuum. Meanwhile, later chronotypes (evening types, or “owls”)—who prefer to go to sleep later in the evening and wake up later in the morning—sit at the other end of the continuum. The majority are located in the middle of the continuum (intermediate chronotypes). Interindividual differences in sleep–wake rhythm arise from the interplay of genetic influences and environmental factors (Hur and Lykken, 1998; Vink et al., 2001). The most important environmental factor is sunlight, which is used by an individual's endogenous circadian clock to entrain to the individual's environment and control the daily physiological (sleep–wake) rhythm across 24 h (Roenneberg et al., 2003; Kantermann et al., 2007; Wagner et al., 2012). Studies have demonstrated that melatonin levels occurring in the blood and activity levels peak at different times of the day, depending on whether participants are earlier chronotypes (earlier peaks) or later chronotypes (later peaks) (Mongrain et al., 2004; Vitale et al., 2015). Due to early work and school start times, later chronotypes particularly have to adapt their sleep–wake times to meet social obligations. Specifically, later chronotypes are required to wake up on schedule, even though they still need to sleep (Kühnel et al., 2016). As a result, later chronotypes' actual sleep–wake times and their biologically preferred sleep–wake times diverge, causing a circadian misalignment (Wittmann et al., 2006). Consequently, later chronotypes need more self-regulatory resources to manage everyday life activities, as they are more likely to be compelled to live against their biological rhythm, which requires greater self-regulatory efforts daily (Kühnel et al., 2018b).

We argue that circadian misalignment would make later chronotypes especially dependent on high-quality sleep. Indeed, Schmidt et al. (2007) and Guarana et al. (2021) summarized and integrated the findings on sleep's role in “inhibit[ing] impulses and overcoming temptations”—an important aspect of self-regulation. They concluded that in this context, individuals' chronotypes should be considered an important variable of interindividual difference. Kühnel et al.'s (2016) findings are in line with this idea; they reported that people who experience a greater circadian misalignment (compared to people who experience less circadian misalignment) procrastinate more the next day when their sleep quality during the night is low. These findings suggest that some people need more resources for self-regulation in everyday life; therefore, they tend to be more dependent on good-quality sleep (Kühnel et al., 2016). Taken together, we propose that later chronotypes, compared to earlier chronotypes, are more dependent on good-quality sleep at night to prevent procrastination at work. Therefore, the second hypothesis of this study is that the chronotype moderates the negative relationship between day-specific sleep quality and procrastination the next day. This negative relationship is stronger for later chronotypes (evening types) compared to earlier chronotypes (morning types).

### Shift to DST, Sleep–Wake Rhythm, and Procrastination

Later chronotypes' greater dependence on good-quality sleep would be even more pronounced under challenging environmental circumstances that aggravate their circadian misalignment. Once a year, many countries around the world switch from standard to summer time (DST) on the last Sunday in March, when the local clock time is set to 1 hour later. The shift to DST means local clock time will be, for example, 3:00 a.m. (DST) instead of 2:00 a.m. (standard time). Kantermann et al. (2007) showed that the entrainment of an individual's endogenous circadian clock to the individual's environment is disrupted by the shift to DST, as the circadian clock is synchronized with the “sun clock” (day/light–night/dark–cycle), which does not change. Given that an individual's circadian clock does not adjust to the new local clock time, but social obligations such as work and school start times follow the new local clock time, the shift to DST potentially induces and/or aggravates a mismatch (circadian misalignment) between individuals' preferred sleep–wake rhythms (chronotype) and their actual sleep–wake times. This should especially be the case for later chronotypes compared to earlier chronotypes, as local clock time is advanced and thus in contrast to what later chronotypes would prefer (Kantermann et al., 2007; Lahti et al., 2008; Allebrandt et al., 2014). We argue that the shift to DST aggravates circadian misalignment, which increases the need for self-regulatory resources, especially for later chronotypes (Kantermann et al., 2007; Kühnel et al., 2018b). The increased need for self-regulatory resources results in later chronotypes depending even more on sleep quality and its replenishing effect (Wagner et al., 2012; Kühnel et al., 2018b), to prevent procrastination the next day (Kühnel et al., 2016). In other words, the shift to DST would increase later chronotypes' dependence on good-quality sleep to prevent procrastination the next day. Accordingly, we propose the third hypothesis for this study: the shift to DST increases later chronotypes' dependence on good-quality sleep, to prevent procrastination the next day. Following the shift to DST (compared to before the shift to DST), later chronotypes are even more dependent on good-quality sleep to prevent procrastination the next day. In technical terms, the chronotype and shift to DST jointly moderate the relationship between day-specific sleep quality and procrastination the next day.

## Methods

### Sample and Procedure

One hundred and one full-time employees from companies in diverse industries across different regions in Germany participated in this repeated-measures study. Data were collected by one of the co-authors as part of her master's thesis. Sixty-seven individuals from the convenience sample were women, and the participants' age ranged from 20 to 66 years (Mean age = 48 years; *SD* = 9). Participants had about ~10 years of professional experience in their current organization, and they worked 40.24 h on average per week. Blue-collar jobs (with mainly physical work) were represented at 28.7%, while 64.2% of the participants mainly did office work (so-called white-collar jobs).

Only non-shift workers and employees who worked at least 30 h per week were requested to participate. To motivate employees to participate in the study, we offered participants individual evaluations of their chronotypes, as well as feedback based on the results of the study. All employees who provided informed consent to participate received a paper-and-pencil questionnaire booklet by mail. First, they completed a general questionnaire (**t0**) before the working week starting with Monday, 23^rd^ of March about their sociodemographic characteristics and chronotype. Then, they were asked to answer identical questionnaires on the Monday, 23^rd^ of March before (**t1**) and the Monday, 30^th^ of March following (**t2**) the shift to DST (shift from Saturday, 28^th^ of March to Sunday, 29^th^ of March). At each survey point, the participants were advised to answer the questions immediately after their workday; they were also reminded by e-mail or *via* SMS to answer the questionnaires at the given time. Following the completion of the entire questionnaire booklet, the participants were requested to return the booklets by mail (free of charge).

Of the 157 questionnaires that were mailed to the participants, 130 were returned (82%). Due to incomplete responses (e.g., answering only one of the day-specific questionnaires or failing to complete the general questionnaire) and participants not completing the questionnaires at the instructed time (e.g., belatedly completing the questionnaire booklet all at once), data from 22 participants were excluded. In addition, seven participants were excluded due to externally determined sleep hours on days off, which is one of the exclusion criteria of the Munich Chronotype Questionnaire (MCTQ, see Measures Section). The final sample comprised 101 employees (completion rate: 64%).

### Measures

#### General Questionnaire (t0)

##### Chronotype

We used the MCTQ (Roenneberg et al., 2003) to assess the participants' chronotypes. The MCTQ assesses the responder's typical sleep times on workdays and work-free days based on their sleep onset and sleep offset (separately for workdays and work-free days). The MCTQ chronotype is determined by calculating the midpoint between sleep onset and offset on free days (including a correction to balance an increased need for sleep due to the accumulated sleep deficit during the workweek) (Roenneberg et al., 2012). Higher values indicate a later chronotype—that is, a later midpoint of sleep on work-free days. For example, if a person's sleep onset on work-free days is at 1:00 a.m. and sleep offset is at 11:00 a.m., the midpoint of sleep on work-free days is at 6:00 a.m.; therefore, the person's chronotype value would be six. For individuals who do not report *unrestricted* sleep times on work-free days, biologically preferred sleep–wake rhythm (chronotype) cannot be calculated with the MCTQ. Exclusion criteria are when respondents use an alarm to wake up on work-free days, or when their naturally occurring sleep on work-free days is prematurely terminated (externally) because of small children or pets requiring attention.

#### Day-Specific Questionnaires on the Monday Before (t1) and the Monday After (t2) the Shift to DST

##### Sleep Quality

We used a single item derived from the Pittsburgh Sleep Quality Index (Buysse et al., 1989) to assess day-specific sleep quality (i.e., “How do you evaluate this night's sleep?”). This item has been used successfully in studies assessing day-specific sleep in the morning (Sonnentag et al., 2008; Hülsheger et al., 2015). Participants rate their overall sleep quality on a 5-point Likert-type scale (ranging from 1 = very poor to 5 = excellent).

##### Procrastination

Day-specific procrastination was assessed using six items from the Tuckman Procrastination Scale (Tuckman, 1991) that were slightly modified to assess day-specific procrastination (Kühnel et al., 2016). Example items are “Today, I needlessly delayed finishing jobs, even when they were important.” and “Today, I promised myself I will do something and then dragged my feet.” The participants' responses were recorded on a 5-point Likert-type scale (ranging from 1 = strongly disagree to 5 = strongly agree). Cronbach's alpha was 0.83 and 0.81 on Monday before the shift to DST (t1) and on Monday after the shift to DST (t2), respectively.

### Statistical Analysis

We used Mplus 8.4 to conduct multilevel analyses, which account for the nested data structure. The nested data structure arises by measuring the variables sleep quality and procrastination more than once, and thus, they exhibit within-person variation (Level 1: within-person level) and between-person variation (Level 2: between-person level). To test our hypotheses, we examined sleep quality as a predictor of procrastination (Hypothesis 1), the joint effect of sleep quality and chronotype as a predictor of procrastination (Hypothesis 2), and chronotype and the effect of “time” (before vs. after the shift to DST) as joint moderators of the relationship between sleep quality and procrastination (Hypothesis 3). The predictor variable “time” is a within-person variable that refers to the two time points that were coded 0 = *Monday before the shift to DST [t1]* and 1 = *Monday after the shift to DST [t2]*. Thus, the predictor variable time depicts intraindividual differences between the Monday before and the Monday after the shift to DST in the dependent variable. Following best practice recommendations by Aguinis et al. (2013) to test cross-level interaction effects, the within-person level variable sleep quality was person-mean centered for all analyses, while the person-level predictor variable chronotype was grand-mean centered.

To predict day-specific procrastination, we specified and compared several nested hierarchical models. In Model 1, we entered the within-person level predictor variable “sleep quality” (Hypothesis 1). In Model 2 we included the person-level predictor variable “chronotype” and a random slope of sleep quality predicting procrastination. The random slope models allow the relationship between sleep quality and procrastination to vary between persons. Model 3, which tested Hypothesis 2, included the interaction term between chronotype and sleep quality as a predictor of procrastination. In technical terms, chronotype was modeled as a predictor of the random slope of sleep quality predicting procrastination; thus, chronotype is a cross-level moderator. In other words, Model 3 tests whether chronotype explains variance in the strength of the relationship between sleep quality and procrastination. Model 4 contained all of the two-way interactions between the predictor variables time, sleep quality, and chronotype predicting procrastination. Model 5 included the three-way interaction between sleep quality, time, and chronotype (Hypothesis 3). In technical terms, chronotype was modeled as a predictor of the random slope of sleep quality × time predicting procrastination.

## Results

### Descriptive Statistics

Table 1 depicts the means, standard deviations, intercorrelations between variables, and intraclass correlation coefficients based on our analyses. We calculated the variance proportions using null models for each day-specific variable. Fifty-nine percent and 91% of the variance of the specific variables procrastination and sleep quality resided on the within-person level, respectively.

**Table 1:** Means, standard deviations, and correlations of variables.

**Variable**	***M***	***SD***	**1-ICC^**b**^**	**1**	**2**	**3**	**4**	**5**
1. Day-specific procrastination	1.34	0.42	0.59	–	−0.25^***^	0.10		
2. Day-specific sleep quality	3.26	0.73	0.91	−0.19	–	−0.23^**^		
3. Time^a^ (before vs. after the shift to DST)	0.50	0.50	1.00	–	–	–		
4. Chronotype	3.45	0.97	–	0.33^**^	−0.03	–	–	
5. Age	41.94	13.08	–	−0.28^**^	0.10	–	−0.46^***^	–
6. Gender^c^	0.66	0.48	–	0.00	−0.06	–	−0.05	−0.03

a *0 = Monday before the shift to DST; 1 = Monday after the shift to DST. Correlations above the diagonal are day-level (within-person) correlations (N = 202). Correlations below the diagonal are person-level (between-person) correlations. To obtain person-level correlations, day-level data were aggregated (N = 101). To obtain day-level correlations, procrastination and sleep quality were centered around the respective person-mean*.

b *Intraclass correlation (ICC) = ratio of the between-person variance to the total variance, 1-ICC = ratio of the within-person variance to the total variance*.

c *Gender: 1 = female, 0 = male*.

** *p < 0.01*,

*** *p < 0.001*.

The within-person correlation between the day-specific variables procrastination and sleep quality (above the diagonal in Table 1) was significant and negative (*r* = −0.25, *p* < 0.001). To calculate the within-person correlation, the day-specific variables were person-mean centered. The between-person correlations (below the diagonal in Table 1) indicate that procrastination was positively related to chronotype (*r* = 0.33, *p* < 0.01), negatively related to age (*r* = −0.28, *p* < 0.01), and not significantly related to gender (*r* = 0.00, *p* = 0.99). Chronotype was negatively related to age (*r* = −0.46, *p* < 0.001). Table 2 presents the nested models.

**Table 2:** Results of multilevel analyses predicting day-specific procrastination.

	**Null model**			**Model 1**			**Model 2**		
	**Est**	***SE***	***t***	**Est**	***SE***	***t***	**Est**	***SE***	***t***
Intercept	1.344	0.042	32.20^***^	1.344	0.042	32.20^***^	1.344	0.039	34.45^***^
Sleep quality				−0.103	0.039	−2.63^**^	−0.101	0.040	−2.38^*^
Time (before vs. after the shift to DST)^a^									
Sleep quality × Time^a^									
**Level 2 predictor**									
Chronotype							0.153	0.040	3.82^***^
**Cross-Level interactions**									
Sleep quality × Chronotype									
Time^a^ × Chronotype									
Sleep quality × Time^a^ × Chronotype									
−2 × log likelihood		274.506 (3)			267.840 (4)			254.334 (6)	
Δ −2 × log likelihood (*df*)					6.667 (1)^**^			13.506 (2)^***^	
Level 1 intercept variance (*SE*)		0.148 (0.021)			0.138 (0.019)			0.136 (0.025)	
Level 2 intercept variance (*SE*)		0.102 (0.027)			0.107 (0.027)			0.086 (0.025)	
Level 2 slope variance (*SE*)—time^a^									
Level 2 slope variance (*SE*)—sleep quality								0.003 (0.023)	
Level 2 slope variance (*SE*)—Sleep quality × Time^a^									
	**Model 3**			**Model 4**			**Model 5**		
	**Est**	***SE***	***t***	**Est**	***SE***	***t***	**Est**	***SE***	***t***
Intercept	1.344	0.039	34.46^***^	1.325	0.045	29.32^***^	1.323	0.044	30.11^***^
Sleep quality	−0.099	0.044	−2.22^*^	−0.044	0.066	−0.66	−0.051	0.063	−0.82
Time (before vs. after the shift to DST)^a^				0.021	0.048	0.44	0.021	0.048	0.43
Sleep quality × Time^a^				−0.079	0.122	−0.80	−0.087	0.116	−0.75
**Level 2 predictor**									
Chronotype	0.153	0.040	3.82^***^	0.208	0.048	4.29^***^	0.162	0.049	3.32^**^
**Cross-level interactions**									
Sleep quality × Chronotype	−0.043	0.036	−1.20	−0.069	0.044	−1.56	0.064	0.063	1.024
Time^a^ × Chronotype				−0.117	0.054	−2.16^*^	−0.119	0.053	−2.24^*^
Sleep quality × Time^a^ × Chronotype							−0.282	0.112	−2.51^*^
−2 × log likelihood		252.756 (7)			247.650 (12)			241.662 (13)	
Δ −2 × log likelihood (*df*)		1.578 (1)			5.086 (5)			6.008 (1)^*^	
Level 1 intercept variance (*SE*)		0.135 (0.028)			0.126 (0.025)			0.128 (0.027)	
Level 2 intercept variance (*SE*)		0.086 (0.026)			0.086 (0.023)			0.078 (0.023)	
Level 2 slope variance (*SE*)—time^a^					0.003 (0.047)			0.003 (0.045)	
Level 2 slope variance (*SE*)—sleep quality		0.002 (0.033)			0.001 (0.024)			0.001 (0.034)	
Level 2 slope variance (*SE*)—Sleep quality × Time^a^					0.017 (0.051)			0.006 (0.046)	

*Est = Estimate*;

a *0 = Monday before the shift to DST; 1 = Monday after the shift to DST*.

* *p < 0.05*,

** *p < 0.01*,

*** *p < 0.001*.

### Test of Hypothesis 1: The Relationship Between Sleep Quality and Procrastination

Model 1 shows that sleep quality at night significantly and negatively predicted procrastination the next day (estimate = −0.103, *SE* = 0.039, *t* = −2.63, *p* < 0.01). Thus, Hypothesis 1 was confirmed; employees procrastinate less on days following nights during which they sleep better.

### Test of Hypothesis 2: The Moderating Effect of Chronotype on the Relationship Between Sleep Quality and Procrastination

Model 3 tested whether employees with later chronotypes (evening types) were more dependent on good-quality sleep at night to prevent procrastination the next day compared to earlier chronotypes (morning types). Model 2 shows that chronotype significantly predicted procrastination (estimate = 0.153, *SE* = 0.040, *t* = 3.82, *p* < 0.001). There was no significant effect of chronotype as a cross-level moderator on the relationship between sleep quality and procrastination in Model 3 (estimate = −0.043, *SE* = 0.036, *t* = −1.20, *p* = 0.167). Thus, Hypothesis 2 was rejected.

### Test of Hypothesis 3: The Joint Effect of Chronotype, Time (Before vs. After the Shift to DST), and Sleep Quality Predicting Procrastination

**Figure 2:**
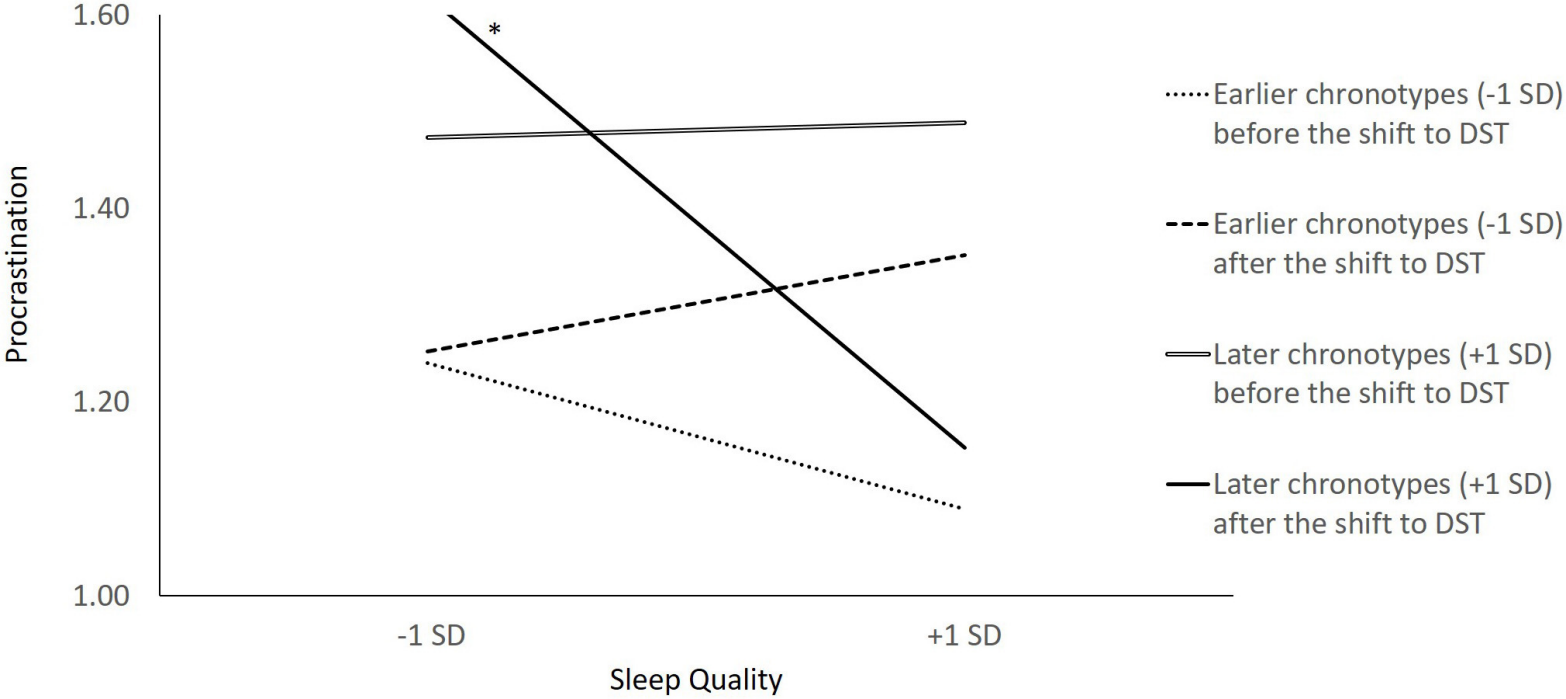
Relationship between day-specific sleep quality and procrastination for earlier (−1 *SD*) vs. later (+1 *SD*) chronotypes before vs. after the shift to daylight saving time.

Finally, we used Model 5 to examine whether the shift to DST increases later chronotypes' dependence on good sleep quality to prevent procrastination the next day. The three-way interaction between time, sleep quality, and chronotype was significant (estimate = −0.282, *SE* = 0.112, *t* = −2.51, *p* < 0.05), and Model 5 fit the data better than the previous model (Δ −2 × log likelihood = 6.008, *df* = 1, *p* < 0.05). We conducted simple slope analyses with Preacher et al.'s (2006) online-based computational tool. We examined the relationship between sleep quality and procrastination for later (+ 1 *SD*) vs. earlier (−1 *SD*) chronotypes for before and after the shift to DST. The simple slope of sleep quality predicting procrastination was significant for later chronotypes after the shift to DST (simple slope = −0.35, *SE* = 0.09, *t* = −3.82, *p* < 0.001). Simple slopes of sleep quality predicting procrastination were not significant for later chronotypes before the shift to DST (simple slope = 0.01, *SE* = 0.08, *t* = 0.15, *p* = 0.88), for earlier chronotypes before the shift to DST (simple slope = −0.11, *SE* = 0.10, *t* = −1.15, *p* = 0.25), or for earlier chronotypes after the shift to DST (simple slope = 0.07, *SE* = 0.12, *t* = 0.62, *p* = 0.53). In other words, the relationship between sleep quality and procrastination was negative for later chronotypes only on the Monday following the shift to DST. Figure 2 demonstrates this relationship. Put another way, only later chronotypes were dependent on good-quality sleep to prevent procrastination on the Monday following the shift to DST. Hence, our findings partially supported Hypothesis 3.

## Discussion

The current study examined the effects of sleep and chronotype on procrastination at work. Moreover, this study considered the effect of the shift to DST as a phenomenon manipulating employees' required sleep–wake times in their everyday work life. We found that day-specific sleep quality predicted procrastination the next day. That is, a lower sleep quality during the night resulted in more procrastination the next day. Further, later chronotypes (evening types) experienced more procrastination than did earlier chronotypes (morning types). Our study's results did not confirm our hypothesis that for later chronotypes compared to earlier chronotypes, sleep quality is more important for procrastination the next day. Our analyses showed that the expected pattern occurred only after the shift to DST. More precisely, after the shift to DST, later chronotypes reported more procrastination during the day when they experienced low-quality sleep the previous night.

Taken together, the results of this study extend the findings on procrastination by providing more insight into the conditions under which procrastination is more likely to occur. We gained a better understanding of the relationship between sleep and procrastination by clarifying the role of chronotype. Furthermore, we identified the shift to DST as a condition that influences different chronotypes' likelihood of procrastination. This study is the first to shed light on the effect of the interplay of sleep quality, chronotype, and the shift to DST on procrastination.

### Sleep Quality and Procrastination

The findings of this study are in line with previous findings that highlight the relevance of sleep for self-regulatory resources to initiate action at work (Baumeister et al., 1998; Kühnel et al., 2016). In particular, we could replicate previous studies' results, showing that on days following nights during which employees had slept better, they procrastinated less (Kühnel et al., 2016). During sleep, the replenishment of the self-regulatory resources is ensured (Baumeister et al., 2000; Barber et al., 2010), but if their replenishment is hampered because of low-quality sleep, people have more difficulties resisting dysfunctional or impulsive actions, and procrastination can occur. Our results underscore the usefulness of the theoretical perspective of self-regulation to understand and predict procrastination. Particularly, sleep quality is a prerequisite to ensure employees' ability to initiate action at work.

### Chronotype and Procrastination

We clarified the role of individually-preferred sleep–wake rhythm (chronotypes) in the relationship between sleep and procrastination. In line with other studies (Díaz-Morales et al., 2008; Digdon and Howell, 2008), our findings showed that later chronotypes procrastinated more than earlier chronotypes. This finding supports our theoretical assumption that later chronotypes may require more resources for self-regulation in everyday life, and consequently, they may be more prone to procrastination in general. Adapting sleep–wake times to their work schedules and coping with one's circadian misalignment (Wittmann et al., 2006) could be possible reasons for later chronotypes needing more self-regulatory resources. Guarana et al. (2021) emphasized the relevance of chronotype and circadian effects in sleep research, as individually-preferred sleep–wake rhythms have been shown to influence impulse inhibition or overcoming temptation. To understand this effect in greater detail, future studies may investigate mechanisms underlying the relationship between chronotype and procrastination.

However, the results did not reveal any differences between chronotypes regarding the importance of sleep quality for procrastination. In our study, there was no difference between chronotypes in their degree of dependence on good-quality sleep. One might speculate that the later chronotypes in our sample might have successfully developed strategies to cope with the increased demands for self-regulation in their daily life. One might also question our assumption that later chronotypes experienced more circadian misalignment in their daily life. It is possible that the later chronotypes in our sample had later and/or flexible work start times and no other social obligations because of which they were less affected by circadian misalignment and thus not more dependent on good-quality sleep compared to earlier chronotypes. Another explanation for our findings is that the distribution of chronotypes in our sample was restricted compared to the distribution of chronotypes in the population. Specifically, earlier chronotypes were considerably overrepresented in our sample, which may have mirrored the reality of earlier and intermediate chronotypes rather than the reality of later chronotypes.

### DST, Chronotype, and Procrastination

Finally, we found that the shift to DST aggravates later chronotypes' dependence on high-quality sleep to prevent procrastination the next day (Culić and Kantermann, 2021). Our findings thus reveal the consequences of the shift to DST, especially for later chronotypes. In particular, our results are thus in line with previous studies, showing the greater dependence of later chronotypes on sleep quality and its replenishing effect (Kühnel et al., 2016; Przepiórka et al., 2019). Regarding procrastination, earlier chronotypes were unaffected by the shift to DST. Our findings support the idea, that a circadian misalignment leads to increased demands for self-regulation in daily life (Kühnel et al., 2018b), and therefore, a greater need for replenishing the resources needed for self-regulation.

### Limitations and Suggestions for Future Research

Later chronotypes were underrepresented in this study's sample, which may have resulted in an underestimation of the true effect of chronotype on the relationship between sleep quality and procrastination at work. Future studies may want to employ a different recruiting strategy to achieve an unrestricted distribution of chronotypes. This may be challenging, however, as especially late chronotypes may refrain from filling in questionnaires immediately after the shift to DST.

The relationship between sleep quality and procrastination might be overestimated due to concurrent measurement of both variables. Yet, studies which assessed sleep quality and procrastination separated in time (sleep quality in the morning and procrastination at the end of the work day; sleep quality before the shift and procrastination at the end of the shift) found within-person correlations between sleep quality and procrastination that were of similar size as the within-person correlation we found [*r* = −0.19 in Kühnel et al. (2018a); *r* = −0.22 in Kühnel et al. (2016); *r* = −0.25 in the current study], mitigating concerns that the relationship found is solely due or strongly inflated due to concurrent measurement.

Measuring self-regulatory resources would allow to explicitly test our theoretical assumption that greater circadian misalignment increases the need for self-regulatory resources, especially for later chronotypes, to prevent self-regulatory failure. Assessing self-regulatory resources would allow researchers to investigate whether dealing with the shift to DST—and thus dealing with circadian misalignment—depletes self-regulatory resources. Future research should examine self-regulatory resources using questionnaires and/or other approaches that assess the availability of self-regulatory resources such as the Stroop test (Kuhl and Kazén, 1999).

A comparison between countries that shift to DST would also be an interesting topic for future research. A question arises whether the current findings are generalizable to other countries that shift from the standard time to DST, or whether this effect is mitigated in countries with a napping culture (i.e., Spanish *siesta*), such as Spain.

Further, the effect of the shift from DST to standard time in autumn may also be an interesting research direction. Researchers could investigate whether later chronotypes, who experience negative effects of the shift to DST, have greater benefits when shifting to standard time. Such studies would provide valuable insights into the consequences of a *reduction* of misalignment, as well as its potential benefits for different chronotypes.

At last, a study with a follow-up design would further extend our findings. In particular, it would be useful to investigate whether the effects of the shift to DST reduce soon after the shift to DST or whether they last longer, maybe even throughout the DST period. A previous study has shown that chronotypes differ in terms of how soon they adapt to DST (Kantermann et al., 2007)—later chronotypes may take longer to adapt to the new local clock time.

### Practical Implications

The results of our study offer ways to reduce procrastination, specifically concerning sleep quality and the shift to DST. Various researchers, including Guarana et al. (2021), Kühnel et al. (2021), Ohayon et al. (2017), and Schmidt et al. (2007) have offered relevant recommendations for improving sleep quality. They propose educational and behavioral approaches, as well as employing influential behaviors by authority figures and role models (Kühnel et al., 2021). Moreover, organizations should also become role models in this regard. One useful approach for organizations is to offer individually-adjustable working hours for different chronotypes to foster good sleep. A trend toward concepts like flex time has already emerged in recent years. Additionally, leaders can foster their employees' sleep by communicating its importance; they can also demonstrate it by taking care of their own sleep.

It is important to consider that later chronotypes in particular not only experience the negative effects of typical work time schedules but also are more affected by the shift to DST. A chronotype-specific training to promote healthy sleep practices and sleep hygiene should be offered at least once a year—ideally, immediately before the shift to DST.

Finally, our findings contribute to the debate around whether DST should be abolished. Existing evidence indicates that the shift to DST does not offer energy-saving benefits (e.g., Aries and Newsham, 2008; Roenneberg et al., 2019), and our results suggest that doing away with the shift to DST could eliminate adverse consequences for later chronotypes.

## Data Availability Statement

The raw data supporting the conclusions of this article will be made available by the authors, without undue reservation.

## Ethics Statement

Ethical review and approval was not required for the study on human participants in accordance with the local legislation and institutional requirements. The patients/participants provided their written informed consent to participate in this study.

## Author Contributions

JK and BZ made substantial contributions to the conception and design of the study. BZ collected the data. TM and JK analyzed and interpreted the data and drafted the manuscript and revised the manuscript for important intellectual content. All authors contributed to the article and approved the submitted version.

## Conflict of Interest

The authors declare that the research was conducted in the absence of any commercial or financial relationships that could be construed as a potential conflict of interest.

## Publisher's Note

All claims expressed in this article are solely those of the authors and do not necessarily represent those of their affiliated organizations, or those of the publisher, the editors and the reviewers. Any product that may be evaluated in this article, or claim that may be made by its manufacturer, is not guaranteed or endorsed by the publisher.

## References

[B1] Aguinis H. Gottfredson R. K. Culpepper S. A. (2013). Best-Practice recommendations for estimating cross-level interaction effects using multilevel modeling. J. Manage. 39, 1490–1528. 10.1177/0149206313478188

[B2] Allebrandt K. V. Teder-Laving M. Kantermann T. Peters A. Campbell H. Rudan I.. (2014). Chronotype and sleep duration: the influence of season of assessment. Chronobiol. Int. 31, 731–740. 10.3109/07420528.2014.901347 24679223

[B3] Aries M. B. Newsham G. R. (2008). Effect of daylight saving time on lighting energy use: a literature review. Energy Policy 36, 1858–1866. 10.1016/j.enpol.2007.05.02134325828

[B4] Barber L. K. Munz D. C. Bagsby P. G. Powell E. D. (2010). Sleep consistency and sufficiency: are both necessary for less psychological strain? Stress Health 26, 186–193. 10.1002/smi.1292

[B5] Barnes C. M. Schaubroeck J. Huth M. Ghumman S. (2011). Lack of sleep and unethical conduct. Organ. Behav. Hum. Decis. Process. 115, 169–180. 10.1016/j.obhdp.2011.01.0099555759

[B6] Baumeister R. F. Bratslavsky E. Muraven M. Tice D. M. (1998). Ego depletion: is the active self a limited resource? J. Pers. Soc. Psychol. 74, 1252–1265. 10.1037/0022-3514.74.5.1252 9599441

[B7] Baumeister R. F. Muraven M. Tice D. M. (2000). Ego depletion: a resource model of volition, self-regulation, and controlled processing. Soc. Cogn. 18, 130–150. 10.1521/soco.2000.18.2.130

[B8] Beutel M. E. Klein E. M. Aufenanger S. Brähler E. Dreier M. Müller K. W.. (2016). Procrastination, distress and life satisfaction across the age range–a german representative community study. PLoS ONE 11, e0148054. 10.1371/journal.pone.0148054 26871572 PMC4752450

[B9] Binnewies C. Sonnentag S. Mojza E. J. (2009). Daily performance at work: feeling recovered in the morning as a predictor of day-level job performance. J. Organ. Behav. 30, 67–93. 10.1002/job.541

[B10] Buysse D. J. Reynolds C. F. Monk T. H. Berman S. R. Kupfer D. J. (1989). The Pittsburgh sleep quality index: a new instrument for psychiatric practice and research. Psychiatry Res. 28, 193–213. 10.1016/0165-1781(89)90047-4 2748771

[B11] Culić V. Kantermann T. (2021). Acute myocardial infarction and daylight saving time transitions: is there a risk? Clocks Sleep 3, 547–557. 10.3390/clockssleep3040039 34842624 PMC8628759

[B12] Díaz-Morales J. F. Ferrari J. R. Cohen J. R. (2008). Indecision and avoidant procrastination: the role of morningness-eveningness and time perspective in chronic delay lifestyles. J. Gen. Psychol. 135, 228–240. 10.3200/GENP.135.3.228-240 18649490

[B13] Digdon N. L. Howell A. J. (2008). College students who have an eveningness preference report lower self-control and greater procrastination. Chronobiol. Int. 25, 1029–1046. 10.1080/07420520802553671 19005903

[B14] Ferrari J. R. (2001). Procrastination as self-regulation failure of performance: effects of cognitive load, self-awareness, and time limits on ‘working best under pressure'. Eur. J. Pers. 15, 391–406. 10.1002/per.413

[B15] Gersick C. J. G. (1989). Marking time: predictable transitions in task groups. Acad. Manag. J. 32, 274–309. 10.5465/256363

[B16] Guarana C. L. Ryu J. W. O'Boyle E. H. Lee J. Barnes C. M. (2021). Sleep and self-control: a systematic review and meta-analysis. Sleep Med. Rev. 59, 101514. 10.1016/j.smrv.2021.101514 34157493

[B17] Hagger M. S. (2010). Self-regulation: an important construct in health psychology research and practice. Health Psychol. Rev. 4, 57–65. 10.1080/17437199.2010.503594

[B18] Howell A. J. Watson D. C. Powell R. A. Buro K. (2006). Academic procrastination: the pattern and correlates of behavioural postponement. Pers. Individ. Dif. 40, 1519–1530. 10.1016/j.paid.2005.11.023

[B19] Hülsheger U. R. Feinholdt A. Nübold A. (2015). A low-dose mindfulness intervention and recovery from work: effects on psychological detachment, sleep quality, and sleep duration. J. Occup. Organ. Psychol. 88, 464–489. 10.1111/joop.12115

[B20] Hur Y.-M. Jr Bouchard T. J. Lykken D. T. (1998). Genetic and environmental influence on morningness–eveningness fn2 fn2Part of the material reported here was presented at the 27th annual meeting of the behavior genetics association. Pers. Individ. Dif. 25, 917–925. 10.1016/S0191-8869(98)00089-0

[B21] Kantermann T. Juda M. Merrow M. Roenneberg T. (2007). The human circadian clock's seasonal adjustment is disrupted by daylight saving time. Curr. Biol 17, 1996–2000. 10.1016/j.cub.2007.10.025 17964164

[B22] Kazén M. Kaschel R. Kuhl J. (2008). Individual differences in intention initiation under demanding conditions: interactive effects of state vs. action orientation and enactment difficulty. J. Res. Pers. 42, 693–715. 10.1016/j.jrp.2007.09.005

[B23] Kim K. R. Seo E. H. (2015). The relationship between procrastination and academic performance: a meta-analysis. Pers. Individ. Dif. 82, 26–33. 10.1016/j.paid.2015.02.038

[B24] Küchler A.-M. Albus P. Ebert D. D. Baumeister H. (2019). Effectiveness of an internet-based intervention for procrastination in college students (StudiCare procrastination): study protocol of a randomized controlled trial. Internet Interv. 17, 100245. 10.1016/j.invent.2019.100245 31080750 PMC6500923

[B25] Kuhl J. Kazén M. (1999). Volitional facilitation of difficult intentions: joint activation of intention memory and positive affect removes stroop interference. J. Exp. Psychol. Gen. 128, 382–399. 10.1037/0096-3445.128.3.382

[B26] Kühnel J. Bledow R. Feuerhahn N. (2016). When do you procrastinate? Sleep quality and social sleep lag jointly predict self-regulatory failure at work. J. Organ. Behav. 37, 983–1002. 10.1002/job.2084

[B27] Kühnel J. Diestel S. Melchers K. G. (2021). An ambulatory diary study of mobile device use, sleep, and positive mood. Int. J. Stress Manag. 28, 32–45. 10.1037/str000021029031757

[B28] Kühnel J. Sonnentag S. (2011). How long do you benefit from vacation? A closer look at the fade-out of vacation effects. J. Organ. Behav. 32, 125–143. 10.1002/job.699

[B29] Kühnel J. Sonnentag S. Bledow R. Melchers K. G. (2018a). The relevance of sleep and circadian misalignment for procrastination among shift workers. J. Occup. Organ. Psychol. 91, 110–133. 10.1111/joop.12191

[B30] Kühnel J. Syrek C. J. Dreher A. (2018b). Why don't you go to bed on time? A daily diary study on the relationships between chronotype, self-control resources and the phenomenon of bedtime procrastination. Front. Psychol. 9, 77. 10.3389/fpsyg.2018.00077 29456519 PMC5801309

[B31] Lahti T. A. Leppämäki S. Lönnqvist J. Partonen T. (2008). Transitions into and out of daylight saving time compromise sleep and the rest-activity cycles. BMC Physiol. 8, 3. 10.1186/1472-6793-8-3 18269740 PMC2259373

[B32] Lay C. H. (1986). At last, my research article on procrastination. J. Res. Pers. 20, 474–495. 10.1016/0092-6566(86)90127-3

[B33] Marcus B. Schuler H. (2004). Antecedents of counterproductive behavior at work: a general perspective. J. Appl. Psychol. 89, 647–660. 10.1037/0021-9010.89.4.647 15327351

[B34] Mongrain V. Lavoie S. Selmaoui B. Paquet J. Dumont M. (2004). Phase relationships between sleep-wake cycle and underlying circadian rhythms in morningness-eveningness. J. Biol. Rhythms 19, 248–257. 10.1177/0748730404264365 15155011

[B35] Ohayon M. Wickwire E. M. Hirshkowitz M. Albert S. M. Avidan A. Daly F. J.. (2017). National sleep foundation's sleep quality recommendations: first report. Sleep Health 3, 6–19. 10.1016/j.sleh.2016.11.006 28346153

[B36] Preacher K. J. Curran P. J. Bauer D. J. (2006). Computational tools for probing interactions in multiple linear regression, multilevel modeling, and latent curve analysis. J. Educ. Behav. Stat. 31, 437–448. 10.3102/10769986031004437

[B37] Przepiórka A. Błachnio A. Siu N. Y.-F. (2019). The relationships between self-efficacy, self-control, chronotype, procrastination and sleep problems in young adults. Chronobiol. Int. 36, 1025–1035. 10.1080/07420528.2019.1607370 31070062

[B38] Ridder D. T. D. Wit J. B. F. (2006). Self-Regulation in Health Behavior. West Sussex: John Wiley & Sons, Ltd. 10.1002/9780470713150

[B39] Roenneberg T. Allebrandt K. V. Merrow M. Vetter C. (2012). Social jetlag and obesity. Curr. Biol. 22, 939–943. 10.1016/j.cub.2012.03.038 22578422

[B40] Roenneberg T. Wirz-Justice A. Merrow M. (2003). Life between clocks: daily temporal patterns of human chronotypes. J. Biol. Rhythms 18, 80–90. 10.1177/0748730402239679 12568247

[B41] Roenneberg T. Wirz-Justice A. Skene D. J. Ancoli-Israel S. Wright K. P. Dijk D.-J.. (2019). Why should we abolish daylight saving time? J. Biol. Rhythms 34, 227–230. 10.1177/0748730419854197 31170882 PMC7205184

[B42] Schmidt C. Collette F. Cajochen C. Peigneux P. (2007). A time to think: circadian rhythms in human cognition. Cogn. Neuropsychol. 24, 755–789. 10.1080/02643290701754158 18066734

[B43] Sonnentag S. Binnewies C. Mojza E. J. (2008). “Did you have a nice evening?” A day-level study on recovery experiences, sleep, and affect. J. Appl. Psychol. 93, 674–684. 10.1037/0021-9010.93.3.674 18457495

[B44] Stead R. Shanahan M. J. Neufeld R. W. (2010). “I'll go to therapy, eventually”: procrastination, stress and mental health. Pers. Individ. Dif. 49, 175–180. 10.1016/j.paid.2010.03.028

[B45] Steel P. (2007). The nature of procrastination: a meta-analytic and theoretical review of quintessential self-regulatory failure. Psychol. Bull. 133, 65–94. 10.1037/0033-2909.133.1.65 17201571

[B46] Tice D. M. Baumeister R. F. (1997). Longitudinal study of procrastination, performance, stress, and health: the costs and benefits of dawdling. Psychol. Sci. 8, 454–458. 10.1111/j.1467-9280.1997.tb00460.x

[B47] Tuckman B. W. (1991). The development and concurrent validity of the procrastination scale. Educ. Psychol. Meas. 51, 473–480. 10.1177/0013164491512022

[B48] van Eerde W. (2003). A meta-analytically derived nomological network of procrastination. Pers. Individ. Differ. 35, 1401–1418. 10.1016/S0191-8869(02)00358-6

[B49] van Eerde W. (2016). Procrastination and well-being at work, in Procrastination, Health, and Well-Being, eds Pychyl T. A. Sirois F. M. (Cambridge, MA: Elsevier), 233–253. 10.1016/B978-0-12-802862-9.00011-6

[B50] Vink J. M. Groot A. S. Kerkhof G. A. Boomsma D. I. (2001). Genetic analysis of morningness and eveningness. Chronobiol. Int. 18, 809–822. 10.1081/CBI-100107516 11763988

[B51] Vitale J. A. Roveda E. Montaruli A. Galasso L. Weydahl A. Caumo A.. (2015). Chronotype influences activity circadian rhythm and sleep: differences in sleep quality between weekdays and weekend. Chronobiol. Int. 32, 405–415. 10.3109/07420528.2014.986273 25469597

[B52] Vohs K. D. Baumeister R. F. (2011). Handbook of Self-Regulation: Research, Theory, and Applications, 2nd edn. New York, NY: Guilford Press.

[B53] Wagner D. T. Barnes C. M. Lim V. K. G. Ferris D. L. (2012). Lost sleep and cyberloafing: evidence from the laboratory and a daylight saving time quasi-experiment. J. Appl. Psychol. 97, 1068–1076. 10.1037/a0027557 22369272

[B54] Wittmann M. Dinich J. Merrow M. Roenneberg T. (2006). Social jetlag: misalignment of biological and social time. Chronobiol. Int. 23, 497–509. 10.1080/07420520500545979 16687322

